# The Role of Failing Autonomic Nervous System on Life-Threatening Idiopathic Systemic Capillary Leak Syndrome

**DOI:** 10.3389/fmed.2018.00111

**Published:** 2018-04-20

**Authors:** Riccardo Colombo, Maddalena Alessandra Wu, Emanuele Catena, Andrea Perotti, Tommaso Fossali, Federico Cioffi, Roberto Rech, Antonio Castelli, Marco Cicardi

**Affiliations:** ^1^Department of Anesthesiology and Intensive Care, ASST Fatebenefratelli Sacco, Luigi Sacco Hospital – Polo ospedaliero, University of Milan, Milan, Italy; ^2^Department of Biomedical and Clinical Sciences, ASST Fatebenefratelli Sacco, Luigi Sacco Hospital – Polo ospedaliero, University of Milan, Milan, Italy

**Keywords:** idiopathic capillary leak syndrome, autonomic dysfunction, heart rate variability, shock, critically ill

## Abstract

Idiopathic systemic capillary leak syndrome (ISCLS) is a rare disease that involves the endothelium and microcirculation, leading to an abrupt shift of fluids and proteins from the intravascular to the interstitial compartment. The consequence of the capillary leakage is a life-threatening hypovolemic shock that can lead to lethal multiple organ dysfunction. The autonomic nervous system (ANS) is central in regulating the cardiovascular response to hypovolemia, but ANS modulation in ISCLS has not yet been investigated. Here, we report ANS activity during acute phase and recovery from a severe ISCLS shock and speculate on the possibility that autonomic mechanisms underlie the pathogenesis of attacks.

## Introduction

Systemic capillary leak syndrome (SCLS) is a rare condition causing recurrent episodes of potentially fatal hypovolemic shock first described by Clarkson et al. (1). Since then, 500 cases have been reported, most of them secondary to malignancies or chemotherapy, 168 out of 500 classified as idiopathic (2). The underlying pathological mechanism is mainly unknown. Several alterations in cytokine pathways and cell-mediated immune response have been associated with SCLS also in its idiopathic form [idiopathic systemic capillary leak syndrome (ISCLS)] (3–6) leading to abrupt endothelial barrier dysfunction (7). The resulting shift of fluids and proteins from the intravascular to the interstitial compartment causes the hypovolemic shock that characterizes disease recurrences.

Recurrences can be divided into three distinct phases: prodromal, leak, and post leak phase (7). Capillary leakage leads to hemoconcentration, hypoalbuminemia, generalized edema, and refractory hypovolemic shock with subsequent complications (8). ISCLS is often misdiagnosed as polycythemia vera or sepsis.

Because of its low incidence, both pathophysiology and therapy of ISCLS are mainly unknown and still controversial. Moreover, the autonomic nervous modulation during ISCLS attacks has never been studied. We report the first description of autonomic nervous system (ANS) modulation during the course of an acute crisis in a patient affected by ISCLS.

### Case Presentation

In January 2017, a 36-year-old man was admitted to our ICU for a life-threatening ISCLS attack. ISCLS was diagnosed 3 months before the admission. Seven months before the admission, he developed bilateral deep venous thrombosis of the femoral and popliteal veins associated with acute kidney injury (AKI) needing renal replacement therapy. After 2 weeks of hospitalization, he was discharged with full recovery from AKI and warfarin treatment. Two months later, he was admitted to the infectious disease ward of its referral county hospital because of weakness, hypotension, and oliguria. Septic shock was hypothesized, and he received third generation cephalosporin. Blood, urine, and stool cultures were all negative, malarial infection was also ruled out. A total body CT scan didn’t reveal remarkable findings. All symptoms cleared in 3 days, and after 1 week, he was discharged from hospital. One month later, the same clinical features recurred, and he was admitted to the medical ward of the same hospital. His conditions worsened rapidly prompting admission to the ICU for severe hypotension. He developed muscle tension without compartmental syndrome and a 10 kg increase in body weight. The diagnosis of SCLS was made at that time according to the triad of sudden circulatory collapse, hemoconcentration, and hypoalbuminemia, and then he was transferred to our ICU. He was given supportive treatment with careful 200 ml boluses of 6% hydroxyethyl starch (HES) 130/0.4 and norepinephrine infusion at 0.1 mcg/kg/min. The critical shock picture resolved within 24 h and after 3 days in ICU, the patient was discharged to the medical ward. Serum proteins electrophoresis revealed a monoclonal IgG k component. After 1 week, he was discharged home on oral theophylline as prophylaxis of recurrences (9).

Two months later, he was admitted again to the emergency department for hypotension, oliguria, hemoconcentration, weight gain, and low back pain. Physical examination revealed mild tenderness of proximal limb muscles, absence of pathological sounds on cardiac and chest auscultation, and unremarkable abdominal findings. A chest X-ray was negative. After 12 h, he worsened and was transferred to our ICU. He had diaphoresis, dizziness, agitation, severe low back pain, thirst, and moderate tenderness of limb muscle compartments. Hemodynamic data are shown in Table 1. A transthoracic echocardiographic examination showed hyperdynamic little ventriculi. We tolerated a permissive hypotension and intravenous 200 ml boluses of 6% HES 130/0.4 were administered when systolic arterial pressure dropped below 70 mmHg or when the patient became unconscious. A total of 1,250 ml of HES and 650 ml of cristalloids were administered intravenously resulting in a net positive fluid balance. Intravenous morphine was administered to provide analgesia. On the second day, his condition rapidly improved, heart rate decreased, while systolic blood pressure and urinary output increased. 600 ml of cristalloids and no HES were infused. On the third day in ICU, his recovery was complete with normal heart rate, further increase of arterial pressure and urinary output, and no need of fluids infusion. During ICU stay, the patient didn’t develop compartment syndrome although plasmatic creatine-phospho-kinase increased significantly. Hemodynamic data, blood sample analyses, and acid–base status during the ICU stay are displayed in Table 1.

**Table 1:** Hemodynamic data, blood sample analyses, and acid–base status during the ICU stay.

Variable	Day 1 (acute phase)	Day 2 (early recovery)	Day 3 (full recovery)
**Hemodynamics**			
μHR (bpm) (SD)	146.6 (1.9)	98.5 (2.8)	68.5 (2.7)
μSAP (mmHg) (range)	75.6 (54.6–91.6)	109 (82.9–136)	145.5 (126.4–156.4)
μMAP (mmHg) (range)	52.4 (37.7–67.7)	88.2 (68.3–109.9)	114.9 (102–129.1)
μDAP (mmHg) (range)	43.5 (31.2–54.6)	76.2 (53.7–93.7)	95 (80.3–114.9)
Urinary output (ml/day)	80	2,600	5,440
Hydroxyethyl starch (HES) (ml/day)	1,250	0	0
Cumulative fluid balance (ml)	1,450	−1,050	−5,170
**Blood analysis**			
Hb (g/dl)	21.9	18	10.5
Ht (%)	64.5	53.4	30.5
RBC (cells/mm^3^)	7,920	6,600	3,840
MCV (fl/cell)	81.4	80.8	81.9
WBC (cells/mm^3^)	59,400	42,470	13,840
Neutrophils (%)	75.6	80.8	81.9
Lymphocytes (%)	18.5	11.8	19
Monocytes (%)	5.3	7.5	8.6
Eosinophils (%)	0.2	0.1	1
PLT (plt/mm^3^)	231,000	246,000	123,000
PTT (INR)	1.62	1.27	1.15
PT (INR)	1.31	1.28	1.2
Fibrinogen (mg/dl)	262	403	605
d-dimer (μg/l)	585	789	388
Urea (mg/dl)	67	102	126
Creatinine (mg/dl)	2.2	3.74	2.1
Glucose (mg/dl)	172	95	97
Na (mEq/l)	134	140	140
K (mEq/l)	5.6	5	3.5
Cl (mEq/l)	111	102	94
Ca (mEq/l)	8	8	7.9
P (mg/dl)	6.8	n.a.	4.1
Mg (mg/dl)	2.2	1.8	2.1
Albumin (g/dl)	2.5	3	3.6
γGT (U/l)	15	15	16
AST (U/l)	17	107	183
ALT (U/l)	5	16	31
LDH (U/l)	279	529	705
CPK (U/l)	129	5,059	10,886
pCHE (U/l)	7,100	6,100	n.a.
Bilirubin (mg/dl)	0.4	0.6	0.6
C-reactive protein (mg/l)	7	42	67
**Acid–base status**			
pH	7.30	7.41	7.49
paO_2_ (mmHg)	219	214	244
paCO_2_ (mmHg)	20	32	43
HCO_3_^-^ (mEq/l)	9.8	20.3	32.8
BE (mmol/l)	−13.1	−3.1	8
Lactate (mmol/l)	3.4	2	0.5
AG (mEq/l)	13.2	17.7	13.2

*μHR is the mean heart rate, μSAP, μMAP, and μDAP are the mean values of systolic, mean, and diastolic arterial pressure of pulsatile beats collected during cardiovascular signal acquisition for heart rate variability analysis. HES, 6% hydroxyethyl starch 130/0.4*.

To evaluate whether ISCLS patients present normal response to shock, we analyzed the ANS activity of this patient during the last ISCLS crisis.

## Materials and Methods

### Data Collection

The patient was connected to an ICU monitor Philips IntelliVue MX800 (Philips Healthcare, Amsterdam, Netherlands). ECG and invasive arterial pressure were recorded on the built-in monitor PC once daily in the morning during a 20 min period, with the patient lying supine and resting. No fluid challenges were administered during the signals collection. All ECG signals were sampled at 500 Hz and arterial pressure at 125 Hz with ixTrend (ixellence GmbH, Wildau, Germany). The arterial pressure wave was resampled offline at 500 Hz with LabChart Pro 8 (AD Instruments, Dunedin, New Zealand).

### ANS Analysis

Autonomic nervous system was studied non-invasively by the spectral analysis of heart rate variability (HRV) with an autoregressive model as previously described (10–14). A spectral com-ponent was labeled as low frequency (LF) if its central frequency was between 0.04 and 0.15 Hz, while it was classified as high frequency (HF) if its central frequency was between 0.15 and 0.5 Hz (13). The HF power was used as a marker of vagal modulation directed to the heart (12). LF may reflect the sympathetic nerve activity and is partially affected by parasympathetic activity (15–17). The ratio LF/HF was considered a marker of the balance between sympathetic and vagal modulation directed to the heart (11, 13). Stationary samples of 500 beats length were chosen for the analysis. The HRV analysis was carried out with Kubios HRV 2.1 (Department of Applied Physics, University of Eastern Finland, Kuopio, Finland, available at www.kubios.com).

## Results

**Figure 1:**
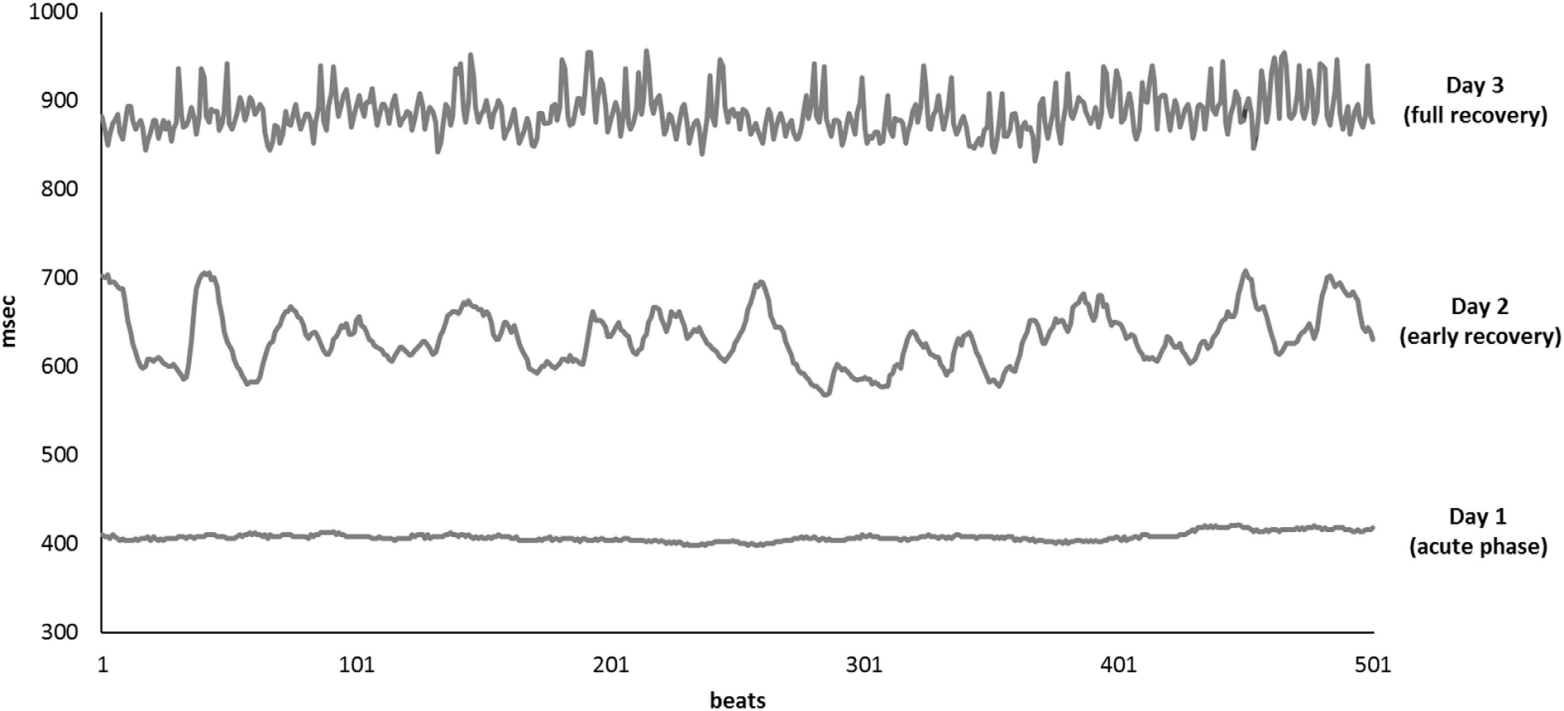
Tachograms of 500 consecutive beats collected on Day 1 (bottom line), Day 2 (middle line), and Day 3 (upper line).

**Figure 2:**
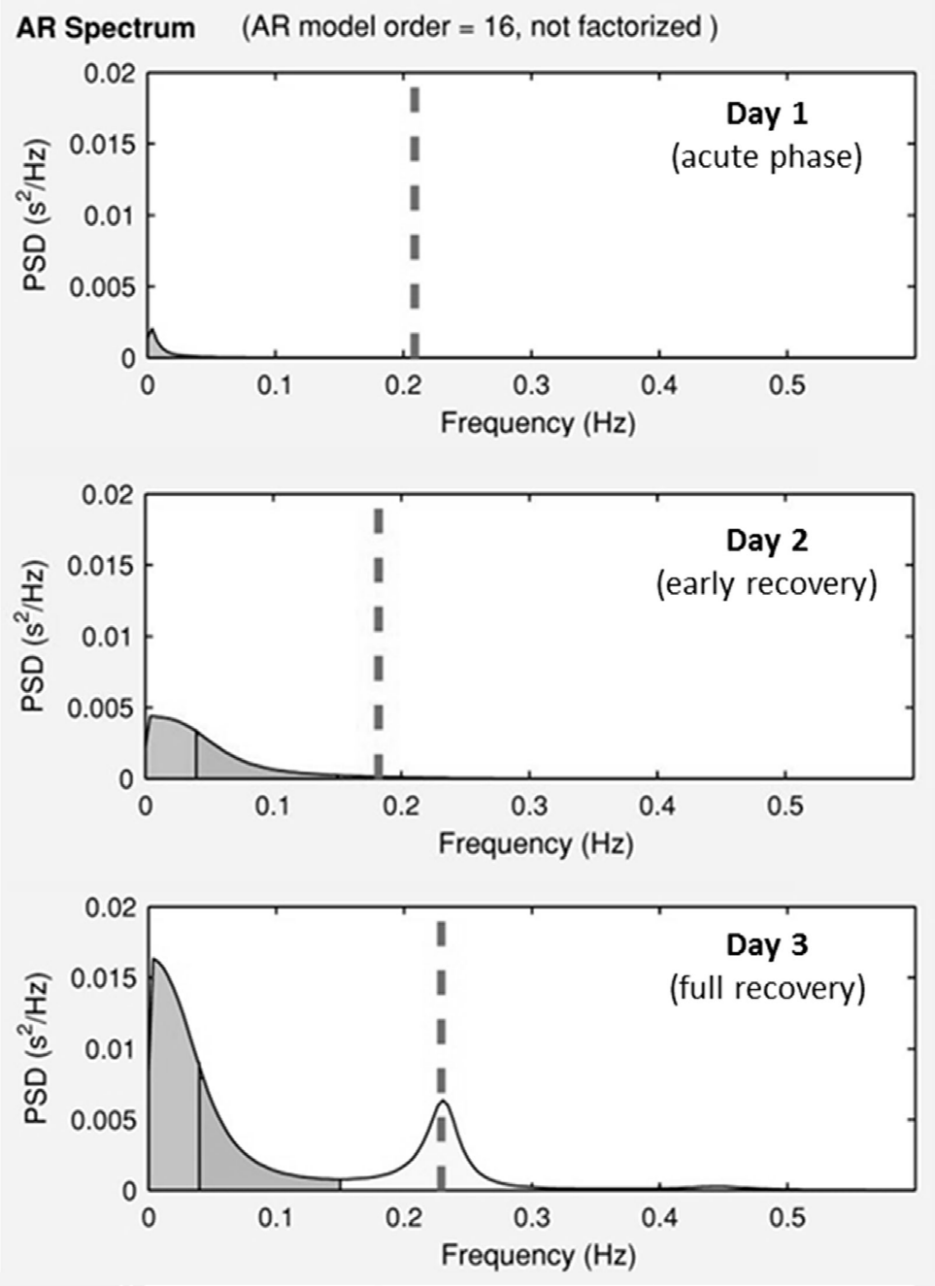
Power spectra of the heart periods during the ICU stay. Day 1, acute phase; Day 2, early recovery; Day 3, full recovery. On the *y*-axis is the power spectra density (PSD), on the *x*-axis is the frequency. Dotted lines represent ECG—derived respiratory rate.

Results of time domain and frequency domain analysis are shown in Table 2. Tachograms and power spectra of HRV during the course of the crisis are shown in Figures 1 and 2.

**Table 2:** Time domain and frequency domain analysis.

Variable	Day 1 (acute phase)	Day 2 (early recovery)	Day 3 (full recovery)
**Time domain results**			
μRR (ms)	409.2	609.8	877.7
STD RR (ms)	5.4	17.3	34.3
RMSSD (ms)	1.2	4.8	24.8
NN50 (count)	0	0	21
pNN50 (%)	0	0	6.2
**Frequency domain results**			
Total power (ms^2^)	25	294	1,120
VLF (ms^2^)	23	159	529
LF (ms^2^)	2	121	273
HF (ms^2^)	0	14	318
LF/HF	8.22	8.87	0.86

*μRR, mean R-to-R interval; STD RR, standard deviation of R-to-R intervals; RMSSD, root mean square of successive R-to-R interval differences; NN50, number of successive intervals differing more than 50 ms; pNN50, percentage of successive intervals differing more than 50 ms; VLF, very low frequency band density; LF, low frequency band density; HF, high frequency band density*.

During the acute phase (Day 1), despite life-threatening hypotension and severe tachycardia, the spectral analysis of HRV didn’t show any oscillatory pattern. Total power was very low, LF band had a density of 12 ms^2^ and HF band of 0 ms^2^, respectively. At early recovery from the ISCLS crisis (Day 2), the power spectrum showed an increase of LF, and a persistently low HF accounting for high LF/HF ratio. At the time of full recovery (Day 3), both LF and HF increased with a prevalence of the HF band, thus the LF/HF ratio was reduced.

## Discussion

Idiopathic systemic capillary leak syndrome is characterized by short lasting, acute episodes of unexplained massive plasma extravasation leading to severe hypovolemic shock. Surprisingly, we found that at the onset of shock, when plasma volume was less than half of the normal (Ht 64 vs 30% in normal conditions), the sympathetic response, normally expected in acute hypovolemia, was absent. The patient’s HRV pattern mimics what is observed in denervated transplanted heart (18, 19) or during complete sympathetic and vagal pharmacological block (11). Sudden changes in circulating blood mass physiologically induce an adaptive autonomic response (20, 21). Mild hypovolemia unloads the arterial and cardiopulmonary receptors decreasing their inhibition over the vasomotor center (22–25). This causes a sympathetic mediated increase of systemic vascular resistance that compensates for the decrease in stroke volume and cardiac output, resulting in mean arterial blood pressure almost unchanged (26). At this stage, spectral analysis of HRV reveals increase of sympathetic and reduction in vagal modulation (27, 28). At the late stage of severe hemorrhage (equivalent to loss of approximately 30% of blood volume), a paradoxical increase of vagal drive may occur leading to a decrease in heart rate, and systemic vascular resistance resulting in a reduction of arterial blood pressure, and possibly to a circulatory collapse (26, 29, 30). An important reduction of ventricles’ volume causes the activation of ventricular mechanoceptors inducing a vagal-mediated bradycardia known as “Bezold–Jarish reflex” (26, 31, 32). The acute phase was characterized by severely compromised hemodynamics. It is also well known that ISCLS patients can develop severe multiple organ dysfunction caused by severe organ hypoperfusion. Our patient was young and without comorbidities, such a condition should guarantee prompt sympathetic response to life-threatening hypovolemia. Instead, adaptive ANS response to shock started with a delay of 24 h. On day 1, there was no detectable oscillatory pattern on HRV spectrum. On day 2, along with patient’s improvement, there was a return of autonomic modulation with a negligible vagal activity and a prevalence of sympathetic modulation. On day 3, when the patient was fully recovered, HRV showed normalization of oscillatory pattern with the prevalence of vagal over the sympathetic activity.

The HRV analysis may have some limitations. LF spectral amplitude is supposed to be influenced by the central volume (33) and HF by heart rate (34). A high interindividual variability in HRV exists in physiologic conditions (13) and during induced hypovolemia (35). On the other hand, however, simulated hemorrhage induced by application of increasing levels of lower body negative pressure until syncope didn’t abolished both LF and HF power spectral densities in healthy subjects (36). Our patient had unexpected flattened HRV spectrum approximating to 0 on the acute phase. Furthermore, there was not interindividual variability because he was the control of himself. He showed a change of his HRV pattern during the recovery from severe sickness. It is important to warn that it is hazardous to generalize from a single case, but nowadays, this is the only one case studied by HRV analysis.

Respiratory and cardiac rhythm act as weakly coupled oscillators with reciprocal influences (37–39). Morris and colleagues demonstrated in an animal model that respiratory and LF cyclic oscillatory component of hemodynamics are coupled at the brainstem level (40). Furthermore, raphè and pontine neurons activity is modulated by baroceptive and vagal activity. In our patient, it is unknown what was the role of baroceptor activity and acute vagal withdrawal in the hemodynamic derangement due to changes of mechanical properties of microcirculation and hypovolemia.

We can interpret the delayed ANS response to shock as pathologic. In acute phases, endothelial cells sieving properties are almost completely disrupted resulting in massive fluid and protein loss. This is due to an endothelial lesion that is purely functional, and it recovers in 24 h. Up to 70% of the plasma mass can shift from intravascular to extravascular compartment (7, 41) and the causes remain unknown. Scattered reports suggest that vascular endothelial growth factor, complement, leukotrienes, angiopoietin 2, interleukin 17, C-X-C motif chemokine 10, endothelial cadherin internalization, and disruption of endothelial junctions may all contribute to the profound hypovolemic state occurring during the extravasation phase (3, 6, 8, 42–47). In this scenario, ANS dysfunction might be considered an additional aggravating factor. As for other changes during acute episodes, distinguishing between pathogenic and compensative response is extremely difficult. The fact that ANS dysfunction reverts in 24 h rules out a damage of neurologic fibers and accounts for a functional response whose negative/positive value remains to be defined.

Nevertheless, we should keep in mind that repeated fluid challenges to increase the central volume can worsen tissue edema, thus reducing peripheral perfusion pressure. Vasoconstrictors such as norepinephrine may be counter-productive because the shock is primarily hypovolemic, thus squeezing further an empty vascular bed doesn’t seem a wise practice.

In conclusion, this case shows that minimizing fluid replacement and amines infusion, with judicious use of colloids to maintain adequate perfusion during the acute phase of ISCLS, allows physicians to avoid further damages. Careful surveillance of potential complications is warranted. Furthermore, our patient showed an unexpected failure of ANS cardiovascular modulation. His HRV was totally suppressed. Further investigation is warranted to assess whether the ANS failure is an epiphenomenon of multiple organ dysfunction during life-threatening attacks or if it might have a role in the pathogenesis of hemodynamic instability during ISCLS crises.

## Ethics Statement

The ethics committee approval is not required because this paper is a case report. The patient provided his consent to publication in accordance with Italian laws.

## Author Contributions

RC and MW analyzed the cardiovascular signals; EC and AP performed echocardiographic assessment; EC, AP, TF, and FC collected all clinical data; RC, FC, and AC assembled the signals acquisition system, calculated the acid base status, and prepared the tables; EC and MC were the clinical decision makers; RC, MW, and RR wrote the first version of the manuscript; MC substantially revised the manuscript.

## Conflict of Interest Statement

The authors declare that the research was conducted in the absence of any commercial or financial relationships that could be construed as a potential conflict of interest.
